# Electronic and Optical Properties of Substitutional and Interstitial Si-Doped ZnO

**DOI:** 10.3390/ma5112088

**Published:** 2012-10-29

**Authors:** Hsuan-Chung Wu, Yen-Chun Peng, Tsu-Ping Shen

**Affiliations:** 1 Department of Materials Engineering, Ming Chi University of Technology, New Taipei 24301, Taiwan; E-Mails: mad-radio@hotmail.com (Y.-C.P.); zoxp3345678@yahoo.com.tw (T.-P.S.); 2 Center for Thin Film Technologies and Applications, Ming Chi University of Technology, New Taipei 24301, Taiwan

**Keywords:** First-principles, DFT + U, Si-doped ZnO, electronic structure, optical property

## Abstract

This study investigates the formation energies, electronic structures, and optical properties of pure and Si-doped ZnO using density functional theory and the Hubbard U (DFT + U_d_ + U_p_) method. The difference in lattice constants between calculated results and experimental measurements is within 1%, and the calculated band gap of pure ZnO is in excellent agreement with experimental values. This study considers three possible Si-doped ZnO structures including the substitution of Si for Zn (Si_s(Zn)_), interstitial Si in an octahedron (Si_i(oct)_), and interstitial Si in a tetrahedron (Si_i(tet)_). Results show that the formation energy of Si_s(Zn)_ defects is the lowest, indicating that Si_s(Zn)_ defects are formed more easily than Si_i(oct)_ and Si_i(tet)_. All three of the Si defect models exhibited *n*-type conductive characteristics, and except for the Si_i(oct)_ mode the optical band gap expanded beyond that of pure ZnO. In both the Si_i(oct)_ and Si_i(tet)_ models, a heavier effective mass decreased carrier mobility, and deeper donor states significantly decreased transmittance. Therefore, the existence of interestitial Si atoms was bad for the electric and optical properties of ZnO.

## 1. Introduction

Wurtzite zinc oxide (ZnO) is a semiconductor with a wide band gap of 3.37 eV and a large exciton binding energy of 60 meV at room temperature [1]. As a result, ZnO has been widely used in photoelectric applications, such as transparent conductive oxides (TCOs) [2], transistors [3], nano-energy-related fields [4], diluted magnetic semiconductors [5], and dye-sensitized solar cells (DSSCs) [6]. 

Doping ZnO with various dopants, such as group-III elements [7,8,9] and group-IV elements [10,11,12,13,14,15,16], is a popular way to improve the performance of ZnO. The group-IV leads to low resistivity and high transmittance in the visible light range for ZnO films doped with Si, Ti, Zr, or Hf, and Si-doped ZnO achieves the lowest resistivity [17]. Given the abundance of silicon on the earth and its non-toxic nature, Si dopant is a promising element for enhancing the electrical and optical properties of n-type ZnO films. Researchers have recently achieved low resistivity and high transmittance in Si-doped ZnO using various fabricating methods [14,15,16]. Clatot [2] fabricated Si-doped ZnO thin films on flexible substrates using pulsed laser deposition at low temperature, and obtained a low resistivity of 8 × 10^−4^ Ω-cm. Nomoto *et al.* [18] indicated that Si dopant can improve the uniformity of resistivity on the substrate surface of AZO:Si thin films by sputtering deposition. These results imply that Si doping in ZnO also has great potential for applications requiring flexible substrates and large area deposition. On the theoretical side, Körner [19] found that the defect of a Si atom substituting for O site in ZnO was unlikely to form compared with the substitution of Si for Zn. Chowdhury *et al.* [20,21] calculated the electronic structure and optical properties of ZnO systems doped with Si. The calculated band gap of ZnO was approximately 0.73 eV, which is significantly smaller than the experimental value of 3.37eV, because of well-known limitations in density functional theory (DFT). Results show that Si dopant reduced the band gap from 0.73 eV to 0.34 eV at 12.5% Si composition, and total reduction in band gap compared to pure ZnO was approximately 0.39 eV.

To obtain a more precise electronic structure for transition-metal oxides, a number of recent theoretical studies [22,23] have investigated the influence of the Hubbard U parameter on the p-orbital electrons of oxygen (U_p_) and d-orbital electrons of the transition metal (U_d_). Sheetz [24] indicated that the correct band gap of ZnO can be obtained using the U values of U_d,Zn_ = 10.5 eV for Zn-3d electrons and U_p,O_ = 7.0 eV for O-2p electrons. The preliminary calculations in the current study show that when U_p,O_ = 7 eV and U_d,Zn_ = 10 eV, the calculated band gap and lattice constants of pure ZnO are in excellent agreement with the experimental values. This study adopts the DFT + U_d_ + U_p_ (U_d,Zn_ = 10 eV and U_p,O_ = 7 eV) method to investigate electronic and optical properties of Si-doped ZnO. This study also investigates the formation energy, band structures, density of states (DOS), and optical properties of Si-doped ZnO.

## 2. Calculation Models and Methods

**Figure 1:**
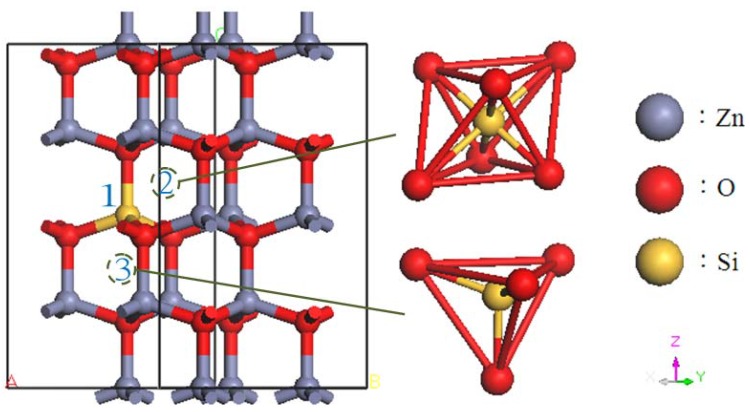
A 2 × 2 × 2 ZnO supercell model containing substitutional and interstitial Si atoms. Gray, red, and yellow spheres represent Zn, O, and Si atoms, respectively.

The ideal ZnO structure is a hexagonal wurtzite structure with two Zn atoms and two O atoms in each primitive cell, and the lattice constants are a = b = 3.249, c = 5.206 Å, *α* = *β* = 90° and *γ* = 120°. This study investigates three types of Si-related defects in ZnO using a 2 × 2 × 2 supercell containing 16 Zn atoms and 16 O atoms. A substitutional doped Si supercell (labeled Si_s(Zn)_) was constructed by substituting one Zn atom with one Si atom (6.25 at%). For interstitial Si doping, one Si atom was embedded into the interspace of the octahedron (labeled Si_i(oct)_) or tetrahedron (labeled Si_i(tet)_). The doping sites of Si atoms, including Si_s(Zn)_, Si_i(oct)_ and Si_i(tet)_, are indicated as 1, 2, and 3, respectively, in Figure 1. In addition, a 2 × 2 × 3 supercell containing 72 atoms was adopted for the Si_s(Zn)_ model (2.78 at%) to compare with the Si concentration of 6.25 at%.

The CASTEP module [25] in Materials Studio 5.5 was employed to calculate the structural, electronic, and optical properties of ZnO using first principle calculations based on the density functional theory. The potentials among ion core and valence electrons were described by ultrasoft pseudopotentials in the Vanderbilt form [26]. The valence electron configurations were 4s^2^3d^10^ for Zn, 2s^2^2p^4^ for O, and 3s^2^3p^2^ for Si. The energy cutoff for a plane wave basis set was 380 eV and a Monkhorst-Pack k-points grid sampling of 4 × 4 × 2 was used [27]. The convergence threshold for self-consistent iterations was set at 10^-6^ eV/atom. The lattice parameters and all the atomic positions were optimized until the force on each atom was below 0.03 eV/Å, the internal stress was below 0.05 GPa, and the displacement of each atom was below 0.001 Å.

**Figure 2:**
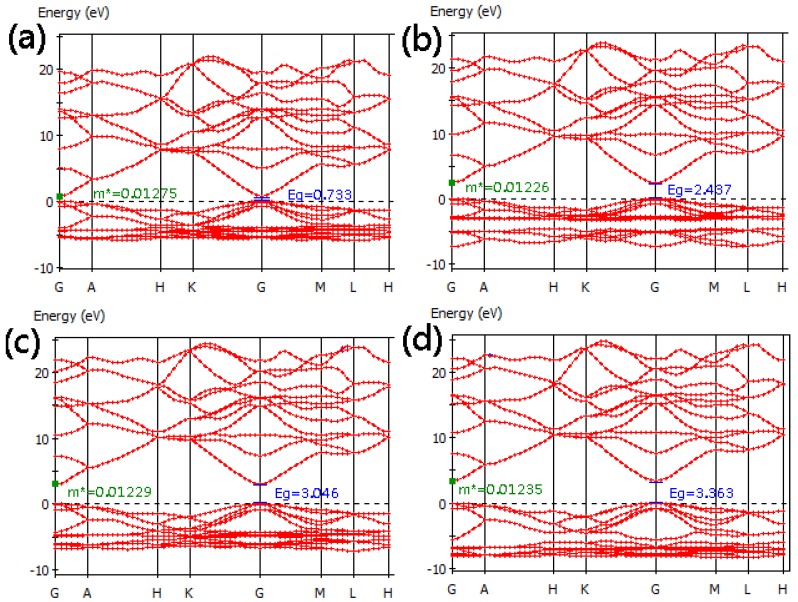
Band structure of ZnO primitive cell for cases A–D.

**Figure 3:**
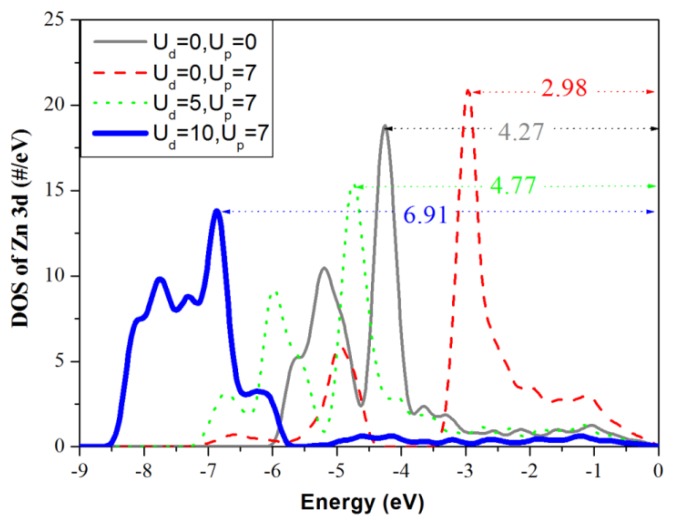
Density of states of Zn-3d as a function of U_d_ and U_p_.

Figure 2 shows the calculated band structures of pure ZnO primitive cells for four (U_d_ + U_p_) cases, including case A (U_d_ = 0, U_p_ = 0) eV, case B (U_d_ = 0, U_p_ = 7) eV, case C (U_d_ = 5, U_p_ = 7) eV and case D (U_d_ = 10, U_p_ = 7) eV. The Fermi level, indicated by a dotted line, is also valence band maximum (VBM) and was set to zero. Figure 2(a) shows that the band gap of ZnO is 0.733 eV (case A), which is consistent with the reported result [20,21], but an underestimate comparison with the experimental value of 3.37 eV, due to the limitation of DFT. Ma *et al.* [22] suggested that for oxide materials the U_p,O_ value of 7 eV is suitable for first principles calculations. Figure 2b shows that the band gap is increased to 2.437 eV for the case B (U_d_ = 0, U_p_ = 7), but this is still far from the experimental value. After applying Hubbard U correction to the Zn-3d states, the band gap increases with an increase in U_d_ in the range from 0 to 10 eV (Figures 2b–d). When U_d_ = 10 eV and U_p_ = 7 eV, the calculated band gap of pure ZnO is 3.363 eV, which is close to the experimental values. Therefore, we adopted U_p_ = 7 eV and U_d_ = 10 eV for this study. The green squares in Figure 2a are the calculated positions of effective masses (m*). It can be seen that the effective mass becomes slightly smaller from case A to case B and is not changed obviously with an increase in U_d_ values (Figures 2b–d). Figure 3 shows the DOS of Zn-3d for cases A-D and zero energy is VBM. It is obviously that the position of the Zn-3d states depends on the choice of the U_d_ and U_p_ values. For case A the maximum peak value of Zn-3d is 4.27 eV below VBM. Under U_p_ = 7 eV condition, the maximum peak of Zn-3d moves toward lower energy level with an increase in U_d_ value. When U_d_ is 10 eV (U_p_ = 7 eV), the maximum peak is 6.91 eV below VBM and average position is around 7.19 eV, which is smaller than the experimental measurement of 7.5 eV [28], but is more accurate than the calculated results [29].

## 3. Results and Discussion

### 3.1. Formation Energy

The formation energies of three different structures of Si-doped ZnO (Si_s(Zn)_, Si_i(oct)_, and Si_i(tet)_) were calculated using a 2 × 2 × 2 supercell to determine the possibility and stability of a defect structure. The formation energies of the Si dopants at substitutional and interstitial sites in ZnO are expressed as follows, respectively:

(1) $$E_{f}(Si_{s})=E_{defect}(Si_{s})–[E_{perfect}(ZnO)–\mu_{Zn}+\mu_{Si}]$$

(2) $$E_{f}(Si_{i})=E_{defect}(Si_{i})–[E_{perfect}(ZnO)+\mu_{Si}]$$

where, *E*_f_(Si_s_) and E_f_(Si_i_) indicate the formation energies of substitutional and interstitial Si defects, *E*_defect_(Si_s_) and E_defect_(Si_i_) are the total energies of the supercell containing substitutional and interstitial Si defects, E_perfect_(ZnO) is the total energy of a perfect supercell of ZnO, and μ_Zn_ and μ_Si_ represent the chemical potentials of the Zn and Si atoms, respectively.

The formation energies depend on growth conditions, which may vary with Zn-rich or O-rich conditions. Table 1 presents a summary of the calculated formation energy for various types of Si-doped ZnO. The E_f_(Si_s_) of O-rich conditions is smaller than the E_f_(Si_s_) of Zn-rich conditions, indicating that the incorporation of Si into ZnO at the Zn site is thermodynamically favorable. The formation energies of Si_i(oct)_ and Si_i(tet)_ are 14.68 eV and 16.56 eV, respectively. These values are much larger than those in the case of substitution, indicating the unfavorable interstitial occupancy of Si in ZnO compared with substitutional sites. Results show that Si atoms preferentially substitute Zn sites, and similar results appear in [19].

**Table 1:** Formation energy, effective mass, and average transmittance of pure and Si-doped ZnO.

	Formation energy (eV)		Effective mass (m_0_)	Transmittance (%)	
	Zn-rich	O-rich		200–400 nm	400–800 nm
**Pure**	–	–	0.22	66	89
**Si_s(Zn)_**	10.79	7.54	0.29	83	86
**Si_i(oct)_**	14.68	14.68	11.49	53	52
**Si_i(tet)_**	16.56	16.56	3.11	53	65

### 3.2. Band Structure

**Figure 4:**
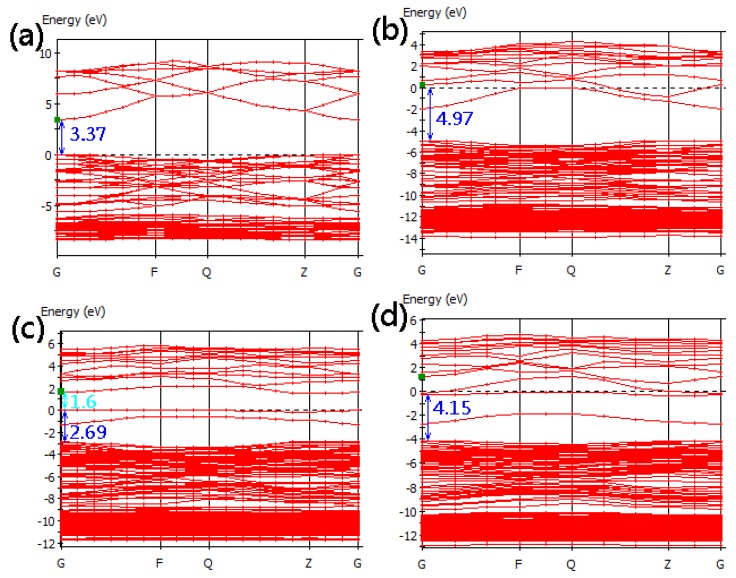
Band structures of Si-doped ZnO for (**a**) pure ZnO; (**b**) Si_s(Zn)_; (**c**) Si_i(oct)_; (**d**) Si_i(tet)_ models.

Figure 4 shows the calculated band structures of pure, Si_s_-doped, Si_i(oct)_-doped, and Si_i(tet)_-doped ZnO supercells. Figure 4a shows that the direct band gap of pure ZnO is 3.37 eV at the *G* point, which is in excellent agreement with experimental values. For the Si_s(Zn)_ in Figure 4b, the Fermi level shifts upward into the conduction band, forming one full-occupied state and one half-occupied state. These results demonstrate a characteristic of the *n*-type conduction. This is because the valence of Si exceeds that of Zn. The optical band gap of 4.97 eV, from the top of the valence band to the Fermi level, is larger than the band gap of pure ZnO. The experimental results also have an increasing trend from pure ZnO to Si-doped ZnO [15]. The Si_i(oct)_ and Si_i(tet)_ models (Figure 4c and Figure 4d) also show *n*-type conductive characteristics, and the optical band gaps of the both models are 2.69 and 4.15, respectively.

In solid-state physics, conductivity is inversely proportional to the effective mass of the free charge carrier, and the effective mass is inversely proportional to the curvature of the energy band. The curvature of the conduction band for the Si_s(Zn)_ model is slightly smaller than that of pure ZnO. However, the conduction bands of both interstitial models become much flatter, resulting in heavier effective masses of electrons in the conduction band. The effective masses of the pure ZnO, Si_s_, Si_i(oct)_, and Si_i(tet)_ models at the G point near the conduction band minimum (green squares in Figure 4) are 0.22, 0.29, 11.49 and 3.11 m_0_ (Table 1), where m_0_ is the rest mass of an electron. It should be noted that the effective masses are different in all directions due to the difference of the curvatures for each band. Even the effective masses at each point are different in the same band in the same direction. Therefore, the Si_s(Zn)_ model has doping in the conduction band with free carriers and a degenerate band situation. The interstitial models have electrons trapped in defect bands and less free carriers. In addition, interstitial Si atoms perturb the conduction band minimum (CBM) severely and make the CBM flatter by the interaction of these bands. The larger effective masses of electrons for both interstitial doping models are related to lower carrier mobility and reduced electrical conductivity.

### 3.3. Density of States

**Figure 5:**
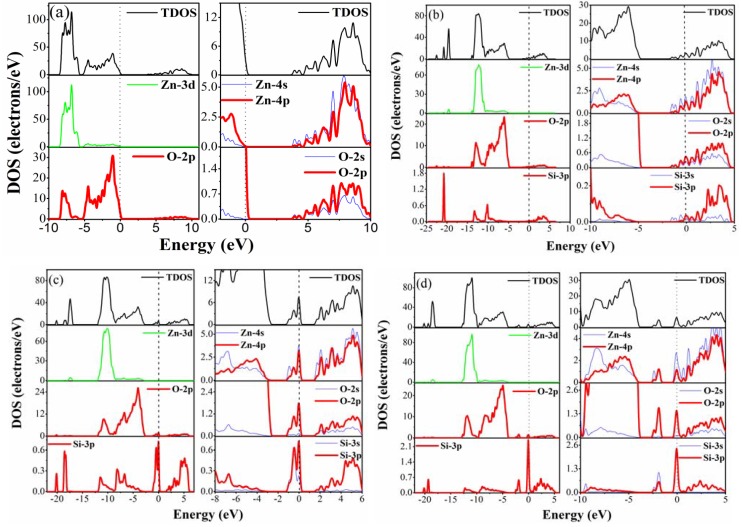
Density of states (DOS) for (**a**) pure ZnO; (**b**) Si_s(Zn)_; (**c**) Si_i(oct)_; (**d**) Si_i(tet)_ models.

To analyze the distribution of each related orbital associated with the constituent elements, Figure 5 shows the DOS for pure and Si-doped ZnO. The Fermi level, indicated by a dotted line, was also set to zero. For the pure ZnO shown in Figure 5a, the lower energy region of the valence band from −8.38 eV to −6 eV shows sharp and narrow peaks, which are mainly composed of the Zn-3d orbital. The upper part of −6 eV to 0 eV consists mainly of the O-2p orbital. The major components of the conduction band include the Zn-4s and 4p orbitals. When replacing one Zn atom with one Si atom, partial of the 3s and 3p orbitals of Si contribute to the occupied states near Fermi level (Figure 5b). These shallow donor states near the Fermi level provide n-type carriers and improve conductivity, which is consistent with the analysis in Section 3.2. Deep donor states appear when Si is doped as an interstitial atom (Figure 5c and Figure 5d). For the Si_i(oct)_ model, the deep states are located at approximately 1.4–2.69 eV above the VBM. For the Si_i(tet)_ model, except for the deep states, the shallow donor states also appear near the Fermi level. Compared with the Si_s(Zn)_ model, the shallow donor states area below the Fermi level (related to the free carrier) of Si_i(tet)_ is smaller. Therefore, the contribution to the conductivity may be less than the Si_s(Zn)_ model.

**Figure 6:**
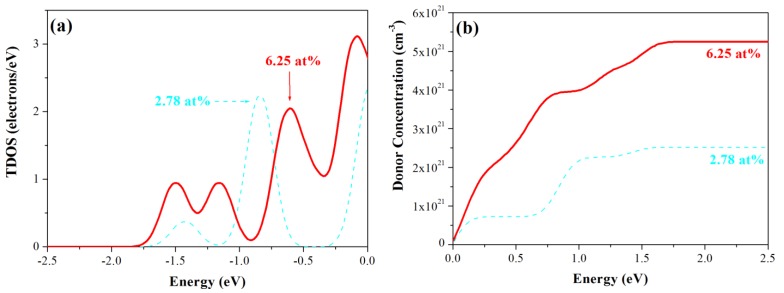
(**a**) Occupied states near the bottom of the conduction band; (**b**) donor concentration under different energy for Si_s(Zn)_-2.78 at% and -6.25 at% models.

The calculated formation energy shows that Si_s(Zn)_ is formed more easily than Si_i(oct)_ and Si_i(tet)_. To compare donor concentration at different Si concentration for the Si_s(Zn)_ models, Figure 6a shows the occupied states of electrons near the Fermi level at 2.78 at% and 6.25 at%. With an increase in Si concentration from 2.78 at% to 3.19 at%, the area of occupied states becomes larger. Differences in the areas at different Si concentrations indicate that the number of electrons entering the conduction band (CB) is not constant. This study calculated the areas from the conduction band minimum (CBM) to the Fermi level to compare the relative number of electrons. Figure 6b shows the donor concentration (cm^−3^) under different excitation energy for Si_s(Zn)_-2.78 at% and -6.25 at%. It can be seen that increasing the Si concentration increases the probability of free electrons entering the CB.

### 3.4. Optical Properties

The optical properties of a material can be described by means of the dielectric function *ε*(*ω*) = *ε*_1_(*ω*) + *iε*_2_ (*ω*). The imaginary part *ε*_2_(*ω*) of the dielectric function is calculated using the following expression [21]:

(3) $$\varepsilon_{2}=\frac{2e^{2}\pi }{{\Omega \varepsilon }_{0}}\underset{k,v,c}{\sum }\;│\;〈\varphi_{k}^{c}│u\cdot r│\varphi_{k}^{v}〉\;{│}^{2}\delta (E_{k}^{c}-E_{k}^{v}-\omega )$$

where *e* is the electronic charge; *Ω* is the unit cell volume; u is the vector defining the polarization of the incident electric field; *ω* is the light frequency; $\varphi_{k}^{v}\;and\;\varphi_{k}^{c}$ are the wave functions of the conduction and valence band, respectively. The real part of the dielectric function *ε*_1_(*ω*) can be calculated from the imaginary part *ε*_2_(*ω*) using the Kramers–Kronig relation. The absorption coefficient *α*(*ω*) can be obtained from *ε*_1_(*ω*) and *ε*_2_(*ω*). The relationship among the absorption coefficient, thin film thickness and transmittance is calculated using the following expression.

(4) $$T=\;{\left(1-R\right)}^{2}e^{-\alpha d}$$

where T is transmittance; R is reflectivity; α is the absorption coefficient; and d is the thickness of the thin film, which was set at 250 nm.

**Figure 7:**
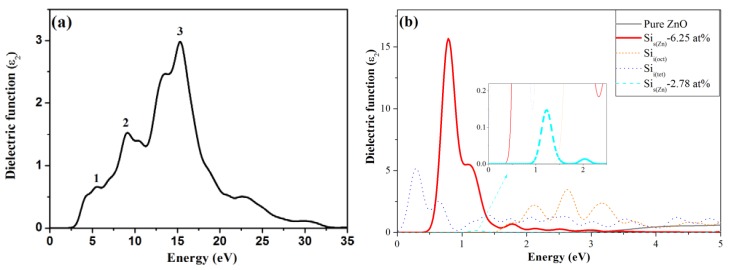
Imaginary part of the dielectric function of (**a**) pure ZnO; (**b**) Si-doped ZnO.

**Figure 8:**
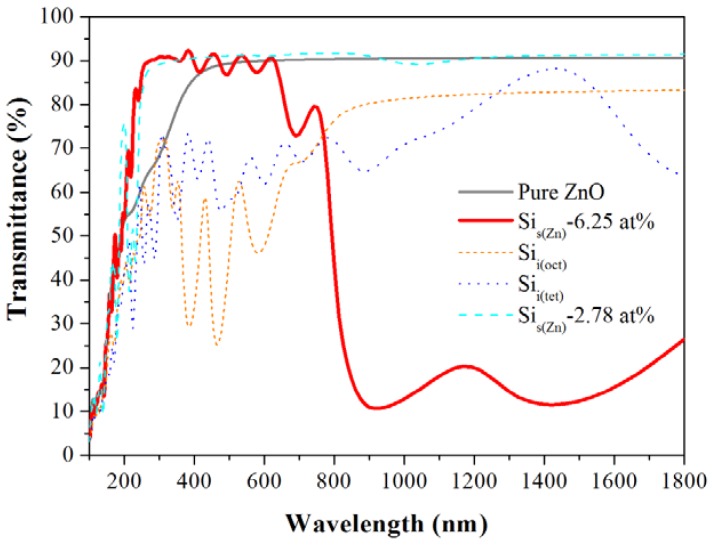
Transmittance spectra of pure ZnO and various Si doping models.

Figure 7 shows the optical properties of pure and Si-doped ZnO as determined through the imaginary part of the dielectric function *ε*_2_(*ω*), representing photo-absorption properties. Figure 8 shows the calculated transmittance spectrum for pure ZnO and various Si doping models. Figure 7a shows the three main peaks in *ε*_2_(*ω*) for pure ZnO, which is in good agreement with experimental results [30]. The average transmittance of pure ZnO is 89% in the visible light region and 66% in the UV region (Table 1). Following the incorporation of Si (6.25 at%), the main peaks of Si-doped ZnO shift toward lower energy (Figure 7b). The previous PDOS analysis in Figure 5b shows that the occupied states between CBM and the Fermi level leads to electron transition behavior at a peak of approximately 0.8 eV. The absorption of the peak decreases the transmittance in the visible light region. However, the enlarged optical band gap increases transmittance in the UV region. Consequently, the average transmittances of the Si_s(Zn)_-6.25 at% model in the visible light and UV regions are 86% and 83%, respectively. For the Si_i(oct)_ model, the three peaks at 2.2, 2.7 and 3.3 eV are mainly contributed from the deep donor states described in Section 3.3. For the Si_i(tet)_ model, the peaks at 0.3 and 0.7 eV are caused by shallow and deep donor states, respectively. The interestitial Si atoms in ZnO significantly decrease the transmittance because of the deep donor states. Therefore, interstitial Si doping produces entirely different characteristics than substitutional Si doping. Comparing with the Si_s(Zn)_-2.78 at% and Si_s(Zn)_-6.25 at% models, at lower concentration of 2.78 at%, the peak at lower energy (the inset in Figure 7b) is weaker than that at 6.25 at%, inducing a slighter absorption in long wavelength region. The average transmittance of Si_s(Zn)_-2.78 at% model in the visible light region is 91% and is higher than that of Si_s(Zn)_-6.25 at% model (Figure 8).

## 4. Conclusions 

In this study we employed DFT + U_d_ + U_p_ to investigate the electronic structure and optical properties of Si- doped ZnO. The calculated band gap and optimized lattice constants were accurately obtained using the Hubbard U values: U_d_ = 10 eV for Zn-3d and U_p_ = 7 eV for O-2p. Results demonstrate that Si_s(Zn)_ has the lowest formation energy, indicating that Zn acts as a preferential dopant site in the crystal lattice. For all Si defects, the Fermi level shifts upward into the conduction band with typical *n*-type conductive characteristics, whereas the optical energy gaps exceed those of pure ZnO except for the Si_i(oct)_ mode. Compared with the pure ZnO model, the shallow donor states in the Si_s(Zn)_ model introduce free carriers into the conduction band, improving conductivity. The transmittance in the visible light region decreases slightly. In both interstitial models, the larger effective masses of electrons are related to a reduction in carrier mobility and electrical conductivity. Additionally, the presence of interestitial Si atoms significantly decrease the transmittance of Si-doped ZnO.
